# Modification of extracellular matrix proteins by oxidants and electrophiles

**DOI:** 10.1042/BST20230860

**Published:** 2024-05-23

**Authors:** Karen C. Yang-Jensen, Sara M. Jørgensen, Christine Y. Chuang, Michael J. Davies

**Affiliations:** Department of Biomedical Sciences, Panum Institute, University of Copenhagen, Copenhagen, Denmark

**Keywords:** atherosclerosis, extracellular matrix, myeloperoxidase, oxidation-reduction, peroxynitrite, post translational modification

## Abstract

The extracellular matrix (ECM) is critical to biological architecture and determines cellular properties, function and activity. In many situations it is highly abundant, with collagens and elastin being some of the most abundant proteins in mammals. The ECM comprises of multiple different protein species and sugar polymers, with both different isoforms and post-translational modifications (PTMs) providing a large variety of microenvironments that play a key role in determining tissue structure and health. A number of the PTMs (e.g. cross-links) present in the ECM are critical to integrity and function, whereas others are deleterious to both ECM structure and associated cells. Modifications induced by reactive oxidants and electrophiles have been reported to accumulate in some ECM with increasing age. This accumulation can be exacerbated by disease, and in particular those associated with acute or chronic inflammation, obesity and diabetes. This is likely to be due to higher fluxes of modifying agents in these conditions. In this focused review, the role and effects of oxidants and other electrophiles on ECM are discussed, with a particular focus on the artery wall and atherosclerotic cardiovascular disease. Modifications generated on ECM components are reviewed, together with the effects of these species on cellular properties including adhesion, proliferation, migration, viability, metabolic activity, gene expression and phenotype. Increasing data indicates that ECM modifications are both prevalent in human and mammalian tissues and play an important role in disease development and progression.

## Introduction

The extracellular matrix (ECM), which surrounds nearly all cells, was once considered to be primarily a scaffold to which cells adhered, with the structure of the ECM determining tissue architecture. However, it is now established that the ECM is multifunctional and a key determinant of cell adherence, migration, proliferation and fate, as well as a determinant of enzyme activity, and cytokine and growth factor availability. Furthermore, there is a growing realization that modifications to the ECM can perturb each of these roles, and that this is of critical importance in a wide range of pathologies. This paper provides an overview of processes that generate ECM modifications (with a focus on unintended alterations), the nature of these changes, and the (known or proposed) role of ECM changes in multiple pathologies, with a particular emphasis on cardiovascular disease.

**Figure 1.**
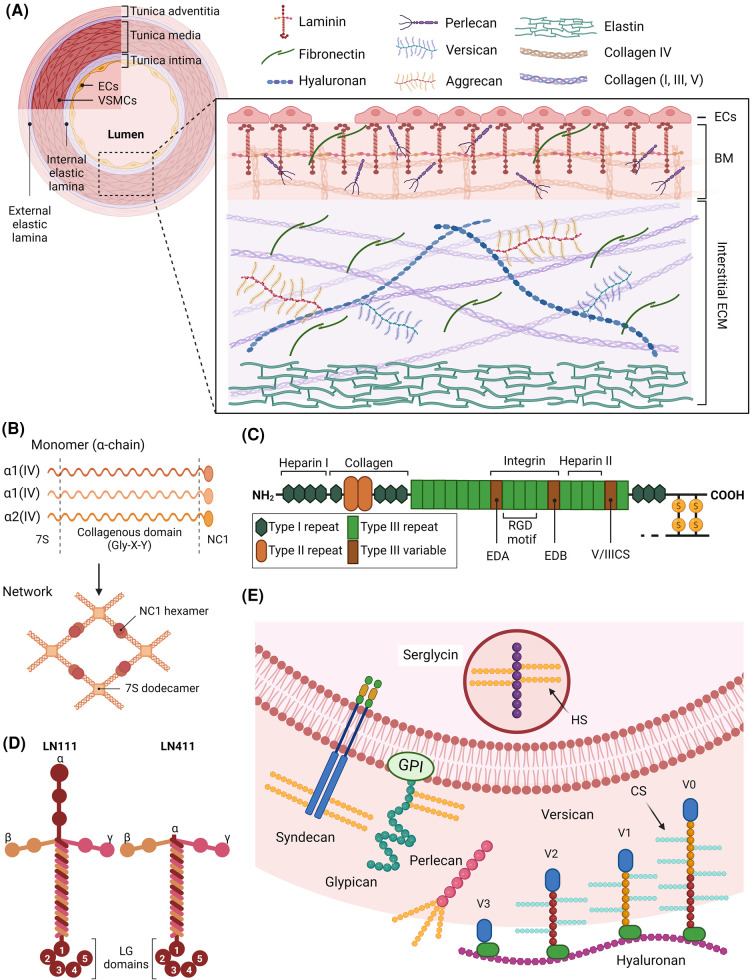
Schematic overview of the arterial extracellular matrix and its components. (**A**) Schematic overview of the three arterial layers, separated by elastic laminae. Each layer is unique in cell and ECM composition. The primary cell type of the intima is endothelial cells (ECs), whereas the media contains several layers of smooth muscle cells (SMCs) while the adventitia contains a mixture of SMCs and fibroblasts. Close up schematic diagram outlining the major ECM components found in the basement membrane (BM) and the interstitial space of a healthy artery (not shown in proportion). (**B**) Structure of collagen (Col) IV monomers and network. Four Col IV (α1)2α2 heterotrimers assemble to dodecamers at their N-terminal 7S domains, while the C-terminal non-collagenous (NC)1 domains forms hexamers of two heterotrimers. (**C**) Diagram of cell-derived fibronectin (FN) monomer including the two extra domains A and B (EDA and EDB respectively) in addition to a variable sequence (known as V or IIICS sequence) arising from alternative splicing. Important FN binding partners and their binding sites are shown, in addition to the intermolecular dimer cysteines at the C-terminal. (**D**) General structure of laminins (LNs). LN α–β–γ chains (indicated by numbers) trimerise via the coiled-coil domains to form the long arm. The C-terminal LG domain is unique to α chains and interacts with cell-surface receptors along with N-terminal globular domains. LN111 and LN411 are shown here highlighting the variations of α chains that can give rise to several other isoforms of LN. (**E**) Subcellular location and structure of several heparan sulfate (HS) proteoglycans (PGs) and four isoforms of versican (V0–V3) attached to hyaluronan (HA) as an example of chondroitin sulfate (CS) PGs. Created by the authors using Biorender.com.

The ECM consists of >300 components that can be grouped into fibrous proteins (e.g. collagens, elastin), proteoglycans (PGs, e.g. versican, perlecan, aggrecan) with attached glycosaminoglycan chains (GAGs), as well as glycoproteins (e.g. fibronectin (FN), laminins (LNs), nidogen). These (glyco)proteins can be further divided into structural proteins and more dynamically expressed matricellular proteins, which regulate interactions between cells and the ECM [1]. A large number of additional matrisome-associated proteins participate in ECM homeostasis, remodeling and modification. These include proteases (e.g. matrix metalloproteinases, MMPs), growth factors, cytokines, proteins that act as scaffolds in ECM assembly) and enzymes involved in protein cross-linking (e.g. peroxidasin, transglutaminases and lysyl oxidases (LOXs)). The composition of the ECM varies across tissues allowing for distinct structures and function. Even within the vasculature, the ECM varies with vessel type and region [2]. The interstitial ECM of the arterial media and adventitial layers is rich in collagens I and III, elastin, FN and PGs. In contrast, arterial basement membranes (BM) are comprised mainly of LNs, collagen IV, perlecan and nidogen. While arterial endothelial cells (ECs) rest on a thick BM, the majority of the arterial BM surrounds vascular smooth muscle cells (VSMCs) in the medial layer, and cells in the adventitia (Figure 1A).

Collagens are multidomain proteins that share a triple helical structure comprised of three α-chains as a common structural motif, together with diverse non-collagenous (NC) domains that provide functional diversity. At least 28 subtypes of collagens exist and the range of structures is further increased by the existence of several α-chains and isoforms for each subtype. In healthy arteries, both collagens I and IV are primarily found as (α1)_2_α2 trimers. The amino acid sequence of the collagen triple helix is characterized by glycine (Gly)-X-Y triads, with X and Y usually proline (Pro) or hydroxyproline. This results in rod-shaped triple-helices that are more or less flexible depending on variations and interruptions in the repeat sequence [1]. Many collagens assemble to supramolecular structures in processes requiring interactions with other collagens, ECM components and matrisome-associated enzymes [3]. Collagen I, III and other fibril-forming collagens assemble side-to-side and end-to-end to form long fibrils with high tensile strength. Collagen IV is assembled into a network important for interactions with both cells and other BM components (Figure 1B).

The second major structural arterial wall protein is elastin, which forms complex fibrils that provide elasticity as they can form extended structures many times longer than the original resting structure [4]. Elastin is highly abundant (30–60% of the dry mass of major arteries), though levels decrease with increasing age, resulting in an increased collagen to elastin ratio, loss of arterial elasticity and increased stiffness. Elastin is predominantly synthesized during tissue development, with little generation in adults except at sites of injury. Mature elastin arises from the deposition of the precursor species tropoelastin onto scaffolds of other ECM species (e.g. fibrillins [4,5]) where it self-assembles into globules that are subsequently heavily cross-linked via oxidation of lysine (Lys) residues by LOXs to allyllysine, and subsequent spontaneous condensation to give irreversible cross-linked species [6,7].

LNs and FN are important vascular ECM glycoproteins. Both have domains for interaction with cell integrins and are thus vital for cell-ECM signaling [2]. FN consists of two near-identical monomers linked at the C-terminus by two disulfide bridges. It exists in two isoforms, both of which are found in the ECM. Plasma FN, synthesized by hepatocytes, is essential for wound healing via reaction with platelets and is a major component of fibrin clots. In contrast, cellular FN is produced locally in tissues and typically contains an extra domain A and/or B, which are missing in plasma FN due to exon skipping [8]. FN has multiple ECM binding partners and is vital in matrix assembly and integrity, as well as cell-matrix interactions due to the presence of both an integrin-binding Arg-Gly-Asp (RGD) sequence, and other domains (Figure 1C).

The LNs are heterotrimeric proteins consisting of one α- (LAMA1–5), one β- (LAMB1–3) and one γ- (LAMC1–3) chain (and named on the basis of their composition such that LN111 contains α-1, β-1 and γ-1 chains), that can assemble into at least 16 different isoforms (Figure 1D). They self-associate to form extensive sheets that act as a matrix scaffold, with the C-terminus of the longest (α) chain binding to integrins, α-dystroglycan, heparan sulfates and other ECM molecules including nidogen. The major LN isoforms expressed in arteries are LN411 and LN511 in the BM of ECs, while LN421 and LN521 are present in the BM generated by VSMC. The LNα2 chain has also been shown to be highly expressed by VSMCs in large arteries [9].

The ECM also contains both free, and protein-bound, linear and branched GAGs. The major free GAG is hyaluronan (HA), a linear (non-sulfated) polymer containing up to 25 000 repeating disaccharide units of D-glucuronic acid and *N*-acetylglucosamine linked by β-(1–4) and β-(1–3) glycosidic bonds, resulting in polymers with molecular masses in the range 5–20 000 kDa. The carboxyl groups on the glucuronic acid monomers result in a negatively-charged surface that binds multiple species including versican. These carboxylates together with multiple hydroxyl groups provide abundant (hydrogen bonding) sites for water molecules with this accounting for its hydration and lubricant properties.

Heparan-, chondroitin- and dermatan-sulfates (the major GAGs found in the cardiovascular system), are complex linear chains, of variable length, made up of repeating uronic acid and amino sugars with highly variable substituent patterns, including regions of extensive sulfation and acetylation. The multiplicity and complexity of the substituent patterns provides a huge diversity of motifs involved in many biological functions including protein binding. Heparin (which is chemically related to heparan sulfate) is a special case as it has regions of exceptionally high negative charge density and is not attached to PGs. Heparin has potent anti-coagulant properties, and heparin-binding domains exist in many ECM species [10].

PGs consist of a protein core with covalently attached GAGs. The functions of PGs are diverse given their different locations (intracellular, cell surface and extracellular) and structures (>30 core proteins and 5 types of GAG chains). In general, PGs act as space-fillers in interstitial matrices, are involved in supramolecular assemblies of ECM proteins, regulate cell behavior, and store soluble factors (e.g. cytokines and growth factors). Classification of PGs is based on the type of attached GAGs (Figure 1E). Heparan sulfate PGs are generally closely associated with cell surfaces and include perlecan, syndecans, glypicans and intracellular serglycin. Chondroitin sulfate PGs predominate further away from cells and include versican, aggrecan and biglycan [11]. Perlecan is the major PG in the BM of ECs in the arterial intima, whereas versican is most abundant in the interstitial ECM of the arterial media. The PG content of the intima can increases dramatically after vascular injury or in disease [12].

Atherosclerosis is a chronic inflammatory disease initiated (at least in part) by the accumulation of low-density lipoprotein (LDL) in the intima of medium- and large-sized arteries. Binding of LDL to ECM components, and especially negatively charged GAGs [13], contributes to LDL-retention. LDL becomes oxidized and modified, activating ECs, which attracts leukocytes. VSMCs migrate from the media to the intima and synthesize ECM to form a fibrous cap covering the plaque [13]. Atherosclerosis is thus characterized by progressive accumulation of lipids, ECM components and cells (leukocytes and VSMCs) in the arterial wall. While atherosclerosis is often asymptomatic in its early stages, continued accumulation of material may cause complete or partial occlusion of the vessel and reduce blood flow causing ischemia. Furthermore, some atherosclerotic plaques may become unstable and rupture causing acute thrombus formation (with potential embolization) resulting in acute clinical events such as myocardial infarction or stroke [14].

## Sources of ECM modifying agents

**Figure 2.**
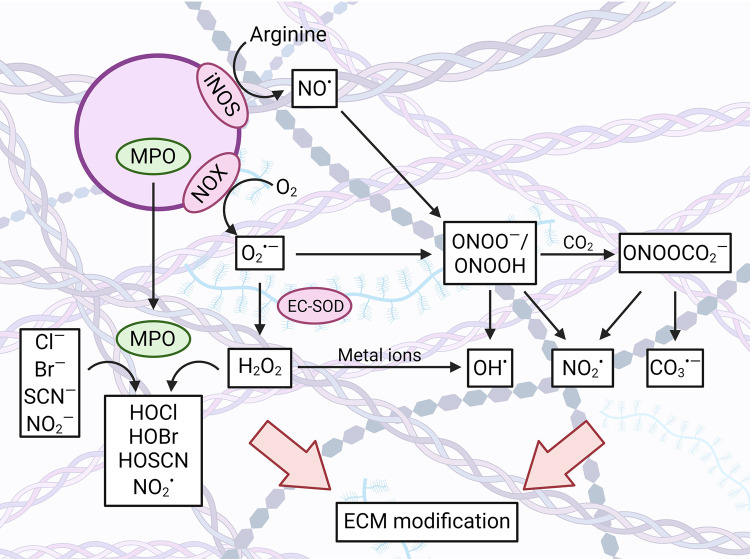
Overview of sources and generation of oxidants. Activation of leukocytes, including monocytes, macrophages and neutrophils, results in the assembly of the NADPH oxidase complex (NOX) at the plasma membrane, which generates O_2_^•−^ that are released into the extracellular space. At the same time, leukocytes release myeloperoxidase (MPO) from intracellular storage granules and the inducible nitric oxide synthase (iNOS) converts arginine to NO^•^. Diffusion controlled reaction between O_2_^•−^ and NO^•^ produces ONOO^−^/ONOOH and in the presence of CO_2_, the adduct species ONOOCO_2_^−^. Decay of this adduct forms CO_3_^•−^ and NO_2_^•^, whereas ONOO^−^/ONOOH forms NO_2_^•^ and HO^•^. Dismutation of O_2_^•−^, either spontaneous or catalyzed by extracellular superoxide dismutase (EC-SOD) enzyme, gives H_2_O_2_. Reaction of H_2_O_2_ with metal ions can produce HO^•^, but H_2_O_2_ may also react with halides (Cl^−^, Br^−^, SCN^−^) or other electron-rich substrates including NO_2_^−^, catalyzed by MPO, to generate hypohalous acids (hypochlorous acid, HOCl; hypobromous acid, HOBr; hypothiocyanous acid, HOSCN) or other products such as NO_2_^•^. Created by the authors using Biorender.com.

Biological systems are continually exposed to oxidants and other modifying agents [15]. This can arise from external agents (cigarette smoke, pollutants, chemicals, high sugar levels, radiation including UV light, oxidized foods, minerals, metal ions) or as a result of metabolic reactions and inflammation [15]. Many endogenous enzymes generate oxidants such as the superoxide radical anion (O_2_^•−^) and hydrogen peroxide (H_2_O_2_) as part of their enzymatic cycles, and these species are also formed by electron transfer chains, particularly those of mitochondria [16,17]. In some cases, these species are by-products of the use of O_2_ as an electron acceptor, whereas in other cases (e.g. NAPDH oxidase enzymes (NOXs), and dual oxidases) O_2_^•−^ and H_2_O_2_ formation is a key component of the innate immune response against pathogens [18,19]. O_2_^•−^ and H_2_O_2_ are part of a large family of reactive species, both radicals and molecular oxidants, often collectively called reactive oxidant species (Figure 2). The use of this term is however discouraged, as it does not discriminate between family members with widely different properties and reactivity [16,17]. This grouping includes highly reactive radicals (species with unpaired electrons, e.g. hydroxyl radical, HO^•^; alkoxyl radicals, RO^•^), poorly reactive radicals (e.g. O_2_^•−^; peroxyl radicals, ROO^•^) and species that induce modifications via two-electron molecular reactions. The latter include H_2_O_2_, other peroxides, peroxynitrous acid/peroxynitrite (ONOOH/ONOO^−^) and hypochlorous acid (HOCl). ONOOH/ONOO^−^ (pK_a_ 6.8) is formed from the reaction of NO^•^ with O_2_^•−^, with the NO^•^ generated by activated leukocytes coming primarily from inducible nitric oxidase synthase (iNOS), and for ECs via endothelial NOS (eNOS) [20]. ONOOH is the more reactive species, and is capable of direct oxidation of targets [20]. However, ONOO^−^ reacts rapidly with CO_2_ (which is in equilibrium with HCO_3_^−^) to give ONOOCO_2_^−^ which undergoes rapid homolysis to give carbonate (CO_3_^•−^) and NO_2_^•^ radicals [20]. O_2_^•−^ and H_2_O_2_ are also generated by the electron transport chains of mitochondria [21], the endoplasmic reticulum [22] and plasma membrane [23], and many (>40; reviewed in [17]) enzymes including uncoupled eNOS [24], xanthine oxidase [25], and LOXs [26]. These species and their interconversion are summarized in Figure 2. The formation of these species has been reviewed in detail elsewhere [16].

Concurrent with O_2_^•−^ and H_2_O_2_ formation, neutrophils, monocytes and some macrophages can release the heme enzyme myeloperoxidase (MPO), while eosinophils release the related enzyme eosinophil peroxidase (reviewed [27,28]). Both enzymes catalyze the formation of hypohalous acids and other oxidants through two distinct catalytic cycles. Both are initiated by two-electron oxidation of the resting Fe^3+^-form of the enzymes to Compound I by H_2_O_2_. In the ‘halogenation cycle’, Compound I is returned to its ferric form by oxidation of halide (Cl^−^, Br^−^ and I^−^) or pseudo-halide (thiocyanate, SCN^−^) anions, giving HOCl, hypobromous acid (HOBr), hypoiodous acid and hypothiocyanous acid (HOSCN) respectively (Figure 2). Alternatively, Compound I can undergo two sequential one-electron reactions, with Compound II as an intermediate, in which electron-rich anions (e.g. NO_2_^−^), radicals (e.g. NO^•^) and aromatic compounds (e.g. phenols and indoles) are oxidized. HOCl and HOBr react with amines to form less reactive, but oxidizing, chloramines (RNHCl) and bromamines (RNHBr), respectively [29,30].

Other diverse non-enzymatic modifications also occur. Glycation and glycoxidation reaction result in advanced glycation end-products, a heterogenous group of non-reversible protein modifications that includes cross-linked species; these accumulate with age and disease [31–33]. These reactions occur with reducing sugars (e.g. glucose) and reactive carbonyls (e.g. glyoxal, methylglyoxal which arise from metabolic intermediates), but can also occur with quinones (present in drugs and natural products) and α,β-unsaturated carbonyls (from pollutants and toxic chemicals, various drugs and also lipid oxidation products. The formation of these species has been reviewed in detail elsewhere [16]. During atherogenesis, LDL oxidation and lipid peroxidation can generate reactive aldehydes including malondialdehyde (MDA), 4-hydroxynonenal and 4-hydroxyhexenal, bifunctional electrophiles that reacts with Cys, Arg, Lys, and other amines potentially forming inter- and intramolecular cross-links (reviewed [34]).

**Figure 3.**
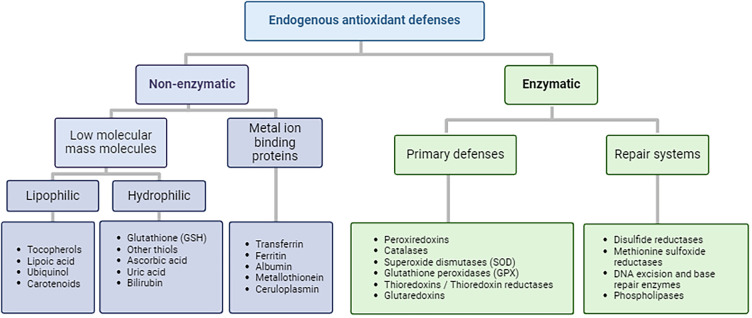
Overview of antioxidant systems. Created by the authors using Biorender.com.

The levels of exogenous and endogenous oxidants is controlled, although not with 100% efficiency, by multiple protective enzymes and repair systems, and to a lesser extent, low molecular mass antioxidants [15,35]. A non-exhaustive list of these systems is provided in Figure 3. Steady-state oxidant levels in biological systems vary markedly, and are situation dependent, but intracellular cytosolic concentrations are typically lower (low nanomolar for H_2_O_2_) than present extracellularly and in some organelles (e.g. endoplasmic reticulum, peroxisomes, Golgi) where H_2_O_2_ may be as high as 10 µM [16,17]. Consequently, the ECM is likely to be exposed to higher levels of oxidants than intracellular proteins, and this situation is compounded by the (typically) lower levels of protective and repair systems present extracellularly (cf. intracellular and plasma concentrations of the key protective thiol compound glutathione, GSH, of 2–10 mM and 2–5 µM, respectively) [36–38].

Oxidants and electrophiles show markedly different reactivity and selectivity, but a common feature is reaction with nucleophiles and aromatic centers (i.e. electron-rich species). Whilst some react with a high degree of specificity with biological molecules, others are poorly discriminate, and modify many targets [16]. Due to their high abundance, proteins are often major targets [39]. Species such as HO^•^ can damage all amino acid chains on proteins, but other agents modify a more limited selection of side-chains, with major targets being the sulfur amino acids cysteine (Cys), methionine (Met) and cystine, aromatic species (tryptophan (Trp), tyrosine (Tyr), histidine (His)) and the amine and guanidine side chains of Lys and Arg, respectively [40]. Unlike the situation within most cell compartments, the concentration of Cys residues (reduced thiol/thiolate form) in most ECM species is rather low. In contrast, large numbers of cystine (disulfide) bonds are present, with many of these playing important structural roles. Thus the Cys/cystine redox balance in the extracellular milieu is dramatically different from most cellular compartments [17,41,42].

Oxygenated products (formed via direct addition reactions, or via the intermediacy of ROO^•^), adducts (particularly with Trp, Tyr, His, Lys and Arg), and cross-linked species are major products. The last of these are typically of low yield due to the ubiquitous presence of O_2_. These reactions and products are reviewed in [40]. The (steady state) concentration of these products depends on the difference between their rates of formation and removal (by specific repair systems or general catabolism). ECM proteins are likely to accumulate particularly high levels [43] due to their abundance, enhanced exposure, poor defense/repair systems, and the long-life times of many ECM proteins (cf. half-lives of >15 years for some collagens and >70 years for elastin [44]). These conditions are difficult to replicate *in vitro*, and some experiments have therefore used high oxidant concentrations over a short period of time to mimic long term exposure to much lower oxidant levels. However this type of approach may not accurately reflect biological reality, as the yield of some products depends not only on the overall oxidant exposure, but also the oxidant *flux* (steady-state concentration), with this being of particular importance for species formed via radical-radical reactions (e.g. di-tyrosine and 3-nitrotyrosine). In some cases, the modifications detected appear to have a clear biological function. In other cases, modification has deleterious consequences. These two divergent scenarios are reviewed below, with a particular emphasis on damaging events and their consequences.

## Physiological ECM modifications

A number of deliberate post-translation modifications (PTMs) arise from cellular and extracellular processing. These include Cys oxidation to disulfides (cystine) during protein folding within the endoplasmic reticulum and mitochondrial intermembrane; this complex process is reviewed in [45,46].

Multiple Lys and Pro side chains on collagens are enzymatically hydroxylated to give 5-hydroxyLys (via lysyl hydroxylase) and 3- and (predominantly) 4-hydroxyPro (via prolyl hydroxylases) within cells before excretion. These transformations are highly efficient and high yield reactions, with 40–45% of the Pro, and ∼25% of the total amino acid complement of collagen I being converted to hydroxyPro [47], which form critical hydrogen bonds that stabilize the collagen triple helix [48]. In contrast, Lys hydroxylation is essential for the attachment of sugars (glycosylation) and the formation of multiple intermolecular covalent cross-links [47,49]. Lys hydroxylation is less extensive than on Pro, and is highly variable between collagen isoforms and occurs at specific sites [47,49]. LOX enzymes generate aldehydes from Lys residues in collagens and elastin with these giving a complex series of cross-links that are essential for correct ECM assembly [49–51].

Transglutaminases catalyze isopeptide bond formation between the ε-amino group of Lys and the side-chain amide of glutamine (Gln) to give (mostly) intermolecular cross-links [52]. Unlike standard peptide bonds, isopeptide linkages are resistant to most proteases and therefore are (relatively) permanent linkages. One of the most well characterized transglutaminases is activated Factor XIII (Factor XIIIa generated from Factor XIII via the removal of the B units) which cross-link fibrin molecules during blood clot formation, with the higher protease resistance of these bonds preventing premature fibrinolysis [53].

Peroxidasin is a member of the heme peroxidase superfamily secreted into the ECM where it mediates cross-linking reactions [54,55]. Unlike other members of the peroxidase family, this enzyme contains additional domains (leucine-rich repeats, immunoglobulin C2 and von Willebrand) that localize this peroxidase to specific ECM sites, possibly via interaction with LNs [56]. The enzyme uses Br^−^ (the reason for this anion being an essential trace element) to generate HOBr. This species oxidizes specific Met residues in collagen NC1 domains to give an intermediate that reacts with a suitably-positioned hydroxyLys residue on another chain to give a sulfilimine (–S=N–) cross-link [54,55]. Peroxidasin or Br^−^ deficiency results in aberrant ECM assembly and early death in *Drosophila* and Zebrafish [55]. Recent data indicate that peroxidasin is essential for EC growth and survival [57] and is involved in cancer progression [58].

Whilst these data suggest that peroxidasin-mediated cross-links are essential for proper ECM synthesis, the mechanisms by which the reactions of HOBr are controlled remain unclear. Bromination of Tyr, an established characteristic reaction of HOBr [29,59], has been detected on collagen IV and other BM-associated ECM proteins, including collagen VI, LNs, fibrillin 1, nidogen-2, and TINAGL1 [60–62]. These modifications may arise from the formation of specific complexes; the proposed binding to nidogen-2 [61] is consistent with the data for peroxidasin interactions with LNs [56], as LN and nidogens form high affinity complexes [63]. Increased bromination of Tyr residues was detected in fibrotic mouse and human lung tissue, where peroxidasin expression was also increased [62]. However, a majority of the identified brominated Tyr residues were also detected in healthy lung tissue [62], consistent with either a physiological role, or alternatively that the formation of sulfilimine cross-links comes at a cost of bystander (and possibly pathological) damage to neighboring ECM molecules; this remains to be clarified.

## Non-physiological and pathological ECM modifications

There is compelling evidence for the association between oxidative events and inflammatory pathologies, including cardiovascular disease. The following sections focus on damage to ECM components and its functional consequences (summarized in Table 1).

**Table 1.** Summary of studies on oxidation and electrophiles on protein-containing ECM components

**ECM source**	**Oxidant/electrophile**	**Structural modifications**	**Functional consequences**	**Reference**
**Collagens**				
Collagen IV from murine renal ECM/human non-diabetic kidney/purified	HOCl, HOBr	Detection of halogenated proteins in renal ECM (diabetic mouse model) ↑ Chlorination of Trp in NC1 hexamers isolated from diabetic renal ECM, human non-diabetic kidney, and isolated protein	↓ Integrin binding to modified ECM and collagen IV ↓ Matrix assembly	[64]
Collagen IV from carotid artery plaque collagen IV/ purified	MDA	↑ MDA-collagen in symptomatic plaques	↓ HUVEC adhesion ↑ HUVEC dysfunction ↑ VCAM-1 expression and ↓ Anti-coagulant proteins in HUVECs	[70]
**Fibronectin**				
Human and bovine pFN HCAEC-ECM	HOCl, HOSCN	Reactivity: HOCl > HOSCN ↓ Antibody recognition and parent amino acids (Met, Trp and Cys) ↑ Protein aggregation Extraction of HOCl^−^ damaged FN from human atherosclerotic plaques	↓ Adhesion and metabolic activity of HCAECs Altered gene expression	[72]
Human pFN	MPO/H_2_O_2_/Cl^−^ or SCN^−^	↓ Antibody recognition of pFN and ↑ HOCl generated epitope with MPO/H_2_O_2_/Cl^−^ No change with MPO/ H_2_O_2_/SCN^−^ SCN^−^ was protective with MPO/ H_2_O_2_/Cl^−^	↓ Cell adhesion and metabolic activity with MPO/Cl^−^ SCN^−^ rescues cell functions	[73]
Human pFN	HOCl, MPO/H_2_O_2_/Cl^−^	Identification/quantification of chlorination and oxidation sites (3-chloroTyr and Trp, His and Met oxidation)	↑ Affinity for heparin ↓ Adhesion of HCASMCs ↑ Proliferation of HCASMCs Altered gene expression	[74]
Human pFN	ONOOH/ ONOO^−^	↑ Protein aggregation, 3-nitroTyr, 6-nitroTrp and di-Tyr ↓ Parent amino acids (Tyr and Trp) Identification of modification sites	↓ Adhesion of HCAECs	[75]
Human pFN	ONOOH/ ONOO^−^	Identification/quantification of modification sites Identification of intra- and intermolecular cross-links (Tyr-Tyr, Trp-Trp and Tyr-Trp)	*** ***	[76]
Human pFN	Oxidized LDL, MDA	Formation of MDA-FN Protein dimerization/aggregation	↓ Response of macrophages (e.g. TNFα, MMP9, PDGF-BB) in response to MDA-FN	[78]
**Elastin**				
Recombinant human tropoelastin	ONOOH/ ONOO^−^	↑ Protein aggregation and 3-nitroTyr ↓ Tyr residues Identification of di-Tyr cross-link		[80]
**Laminins**				
Murine LN111 BME	ONOOH/ ONOO^−^	↓ Antibody recognition of LN111 and parent amino acids (Tyr and Trp) ↑ Protein aggregation, 3-nitroTyr, 6-nitroTrp and di-Tyr	↓ Adhesion of HCAECs	[84]
Murine LN111	ONOOH/ONOO^−^	Identification/quantification of nitration sites	Altered structure of LN polymers ↓ Adhesion of HCAECs	[85]
Murine LN111 BME	HOCl, MPO/H_2_O_2_/Cl^−^	Identification/quantification of modification sites Oxidation of Met and Trp Chlorination of Tyr		[86]
**Proteoglycans**				
HCAEC-derived perlecan	ONOOH/ONOO^−^	↓ Antibody recognition of core protein and HS chains ↑ 3-nitroTyr, carbonyls and protein aggregation	↓ Adhesion of HCAECs ↓ FGF2 binding and FGF2 mediated proliferation (Baf-32 cells)	[88]
rhV3	ONOOH/ONOO^−^, SIN-1	↑ Protein aggregation, nitration of Tyr and Trp, hydroxylation of Tyr and Trp, oxidation of Met, di-Tyr	↓ Adhesion of HCASMCs ↑ Proliferation of HCASMCs ↓ HA binding	[89]
Aggrecan ADAMTS1	HOCl, chloramines	Protein fragmentation Protein aggregation (by irreversible cross-links)	Inactivation of ADAMTS1	[90]
**Complex ECM**				
HCASMCs	ONOOH/ONOO^−^	ECM species over-represented in the pool of modified proteins FN and thrombospondin-1 are particularly heavily nitrated		[69]
HCASMC-ECM	HOCl, MPO/H_2_O_2_/Cl^−^	↓ Antibody recognition of ECM components (FN, collagen IV, LN, versican) ↑ HOCl-generated epitope	↓ Adhesion of HCASMCs ↑ Proliferation of HCASMCs Altered gene expression	[65]
HCAEC-ECM	ONOOH/ONOO^−^	↓ Antibody recognition of ECM components (Collagen IV, FN, LN, perlecan) ↑ 3-nitroTyr	↓ Adhesion of HCAECs Altered gene expression	[67]
HCASMC-ECM	ONOOH/ONOO^−^	↓ Antibody recognition of ECM components (collagen I, collagen III, cFN, LN, versican, CS) ↑ 3-nitroTyr	↓ Adhesion of HCASMCs ↑ Proliferation of HCASMCs ↑ Gene expression of inflammatory mediators and ECM proteins	[68]
Human lung fibroblast ECM Collagen I	HOCl, HOBr	Protein fragmentation (isolated collagen I)	↓ Adhesion and proliferation of human lung fibroblasts to modified ECM ↑ Cytokine production by human lung fibroblasts on modified ECM	[101]
ECM from PFHR9 cells. Healthy and fibrotic mouse lung tissue. Human lung tissue.	HOBr, PXDN/H_2_O_2_/Br^−^	Bromination of Tyr residues in LN and other ECM proteins upon treatment of the ECM from PFHR9 cells with HOBr. Brominated Tyr residues on various BM proteins (LN, collagen IV and VI, fibrillin 1, nidogen-2, TINAGL1) from healthy and fibrotic mouse lungs, and human lung tissue.		[62]

Abbreviations: BME, basement membrane extract; CS, chondroitin sulfate; ECM, extracellular matrix; FGF, fibroblast growth factor; HA, hyaluronan; HCAEC, human coronary artery endothelial cell; HCASMC, human coronary artery smooth muscle cell; HOBr, hypobromous acid; HOCl, hypochlorous acid; HOSCN, hypothiocyanous acid; HS, heparan sulfate; HUVEC, human umbilical vein endothelial cell; LDL, low-density lipoprotein; LN, laminin; MDA, malondialdehyde; MMP, matrix metalloproteinase; MPO, myeloperoxidase; NC, non-collagenous domain; PDGF, platelet-derived growth factor; pFN, plasma fibronectin; PXDN, peroxidasin; rhV3 recombinant human versican isoform V3; TINAGL1, tubulointerstitial nephritis antigen-like 1; TNF, tumor necrosis factor; VCAM-1, vascular cell adhesion molecule-1.

### Collagens

Unlike the specific modifications induced by peroxidasin on collagen IV, that help stabilize the collagen IV network formation in the BM, aberrant reactions may disrupt interactions with other ECM molecules, as well as cells. Collagen IV NC1 hexamers exposed to HOCl lose their ability to reassemble in a dose-dependent manner [64]. Exposure of native ECM synthesized by VSMCs or ECs to either HOCl or MPO/H_2_O_2_/Cl^−^ results in decreased antibody recognition of collagen IV, and damage to the native collagen IV network as evidenced by fragmentation of fibers colocalizing with HOCl-induced epitopes [65,66]. It should be noted that such changes in antibody recognition data need to be interpreted with care as other changes apart from oxidation (e.g. in protein conformation induced by modification of other species) may also result in altered antibody responses. Oxidation and chlorination of two specific Trp-residues (Trp28 of NC1 α1, and Trp192 of NC1 α2 in rats) has been identified on collagen IV NC1 treated with HOCl, and in experimental diabetic rat kidney [64]. Chlorination of Trp1512 of collagen IV α1 has been detected in non-diabetic human kidney [64]. In addition to effects on ECM assembly, collagen IV modification by HOCl or HOBr *in vitro* resulted in decreased binding of α1β1 integrin [64], suggesting that cell-matrix interactions are affected by collagen IV halogenation. It is unclear whether these specific oxidative modifications have a physiological role, are a result of ‘collateral damage’ induced by peroxidasin activity, or arise from MPO activity. Furthermore, it is unclear whether collagen IV halogenation occurs in and contributes to cardiovascular disease.

Antibody recognition of collagen IV in the ECM produced by human coronary artery ECs (HCAECs) is decreased upon exposure to ONOOH/ONOO- at high (100 μM) doses [67], although to a lesser extent than for other ECM components including LN. Interestingly dose-dependent loss of antibody recognition was seen for collagen I and collagen III, but not collagen IV, in ONOOH/ONOO-treated ECM from human coronary artery smooth muscle cells (HCASMCs) [68]. Proteomic analysis to determine sites of nitration has revealed multiple proteins carrying 3-nitroTyr, including collagen VI [69]. However, no other collagen was nitrated indicating that other ECM components are the main targets of ONOOH/ONOO-.

Collagen IV modified by the reactive aldehyde MDA has been suggested to promote atherogenesis. MDA-modified collagen IV is increased in cerebrovascular symptomatic carotid plaques compared with non-symptomatic plaques [70]. Plasma IgG auto-antibodies against MDA-collagen IV correlate with plaque MDA-collagen IV and are associated with risk of myocardial infarction. Furthermore, high levels of plasma auto-antibodies associate with proteases including MMP10 and MMP12, perhaps reflecting a mechanism for removal of modified collagen IV, but this may also be due to aberrant ECM degradation and increased plaque vulnerability to rupture [71]. *In vitro*, MDA-modified collagen IV causes endothelial dysfunction with decreased attachment and anti-coagulant properties as well as increased VCAM-1 expression [70]. The role of MDA-collagen IV and the relevance of autoantibodies in the development of atherosclerosis and other pathologies remains to be clarified.

### Fibronectin

FN colocalizes with HOCl-induced epitopes in human atherosclerotic plaques and is pulled down by antibodies against HOCl-induced epitopes [72]. *In vitro*, antibody recognition of human plasma FN is decreased upon exposure to HOCl or MPO/H_2_O_2_/Cl^−^ in a dose-dependent manner and correlates inversely with HOCl-induced epitopes [72,73]. HOCl- and MPO/H_2_O_2_/Cl^−^-exposure result in dose-dependent formation of 3-chloroTyr from Tyr as well as Met, Trp and His oxidation [74]. The sites of modification differ between reagent HOCl and MPO/H_2_O_2_/Cl^−^, with fewer sites identified with the enzyme system. This may be due to binding of MPO to FN [65], which may direct oxidative events to FN regions close to the MPO active site. This hypothesis is supported by the fact that MPO-mediated modification occurs to a greater extent than with HOCl in the heparin-binding regions of FN. HOCl-induced modification of FN results in increased heparin-binding capacity [74] and affects cell behavior, with HCAECs and HCASMCs cultured on HOCl-modified FN exhibiting altered adhesion, proliferation and gene expression [72,74].

FN is also modified by ONOOH/ONOO- as evidenced by formation of non-reducible high molecular mass aggregates, 3-nitroTyr and (to a lesser extent) 6-nitroTrp at specific sites [75]. The aggregates are likely to be due, at least in part, to di-Tyr, di-Trp and Trp-Tyr cross-links [75,76]. The sites and degree of nitration differ between isolated plasma FN [76] and FN in native VSMC-ECM [69], possibly due to interactions between FN and other ECM components. This may render certain regions of FN inaccessible to the oxidant, while cryptic sites which are not accessible in plasma FN, may be revealed in the extended conformation upon fibril-formation in ECM. FN appears to be a major target of OONOOH/ONOO-, along with thrombospondin-1, in native ECM generated by VSMCs, with these two species being detected with the most sites of nitration [69]. This is further evidenced by a marked dose-dependent loss of parent antibody recognition on ONOOH/ONOO- treatment [68]. FN nitration may be functionally significant, as vascular ECs cultured on ONOOH/ONOO-modified FN exhibit decreased adhesion when compared with native FN [75]. In human atherosclerotic plaques, FN also co-localizes with staining for 3-nitroTyr [75,77].

Exposure of FN to both MDA and oxidized LDL results in the detection (using an MDA-FN antibody) of a species with a higher molecular mass than native FN, consistent with a MDA-induced cross-links [78]. MDA-modified FN has also been detected in human atherosclerotic plaques in both the fibrous cap and core regions, with this colocalizing with oxidized LDL and CD68+ macrophages. Interestingly, MDA-modified FN exhibits significantly altered immunogenicity compared with the native protein. Thus, MDA-FN completely abolishes macrophage secretion of proinflammatory TNFα, platelet-derived growth factor (PDGF)-BB and MMP9 *in vitro*. Unlike auto-antibodies against MDA-collagen IV, auto-antibodies against MDA-FN appear to be atheroprotective, with baseline plasma MDA-FN auto-antibodies being associated inversely with adverse cardiovascular outcomes (myocardial infarction or sudden cardiac death) [78]. Immunization of mice with MDA-FN results in a less inflammatory plaque phenotype and decreased plaque area (69%) [79]. The mechanism behind this effect remain to be elucidated.

### Elastin and fibrillins

Tropoelastin is highly sensitive to ONOOH/ONOO- with this resulting in extensive dimerization, fragmentation and 3-nitroTyr formation [80]. Di-Tyr cross-links (both inter- and intra-protein) have been detected by MS. Colocalization of elastin and 3-nitroTyr has also been detected in human atherosclerotic plaques [80]. Redox-mediated changes to fibrillin proteins, which play a key role in elastin assembly, have also been reported [81] with some of these believed to mimic genetic mutations responsible for Marfan syndrome [82] and aortic aneurysms. In particular redox alterations to structural disulfides in the fibrillins, with formation of either reduced Cys residues or mixed disulfides appear to be important [81]. However, other researchers have reported that other proteins (e.g. smooth muscle cell α-actin and annexin A2) are the major targets of redox changes in Marfan syndrome [83].

### Laminins

LNs are highly susceptible to damage by both HOCl and ONOOH/ONOO^−^ [84–86]. Treatment of LN with reagent HOCl results in the detection by MS peptide mass mapping of oxidation at 30 Met and 7 Trp residues, and chlorination at 33 Tyr residues (with 3 sites also detected as 3,5-dichloroTyr). An additional 8 Met and 10 Trp oxidation sites, and 14 3-chloroTyr, and 18 3,5-dichloro-Tyr sites were detected upon LN exposure to MPO-derived HOCl (i.e. an MPO/H_2_O_2_/Cl^−^ system) [86]. ONOOH/ONOO^−^ treatment of isolated murine LN111 or within a BM extract results in a loss of Tyr and Trp, and formation of 3-nitroTyr, 6-nitroTrp and di-Tyr; these modifications have been shown to be associated with decreased HCAEC adhesion to the modified ECM [84,85]. Treatment of HCAEC-ECM and HCASMC-ECM with ONOOH/ONOO^−^ results in a dose-dependent loss of antibody recognition of LN, and LN colocalizes with 3-nitroTyr in atherosclerotic plaques [67,68].

Human plasma has been shown to contain auto-antibodies against MDA-modified LN [87]. Similar to MDA-FN, autoantibodies against MDA-LN are inversely associated with the risk of developing cardiovascular events. However, unlike for MDA-FN, immunization with MDA-LN exacerbates atherosclerosis development in mice [87].

### Proteoglycans

Both perlecan protein and its attached heparan sulfate are modified by ONOOH/ONOO^−^, with both GAG chain fragmentation and formation of 3-nitroTyr, and protein carbonyls detected [88]. These changes are associated with changes to its biological properties with these manifested as a loss of EC adhesion, diminished binding of fibroblast growth factor 2 (FGF2) and a lack of proliferation of FGF receptor 1c expressing cells [88]. The observed damage is pH-dependent with heparan sulfate chain damage more prominent at pH 6 and 6.5 when compared with 7 and 7.4. In contrast, generation of 3-nitroTyr is enhanced at pH 7 and 7.5. In each case, the presence of CO_2_/HCO_3_^−^ decreased the observed damage [88].

Exposure of HCASMC-ECM to varying concentrations of ONOOH/ONOO^−^ resulted in a dose dependent loss of the versican G1 domain epitope (and other ECM components), concurrent with formation of 3-nitroTyr [68]. When exposed to 100 μM or more ONOOH/ONOO^−^, antibody recognition of versican was reduced by >90%. In contrast, antibody recognition of chondroitin sulfate, the GAG attached to versican, was only reduced significantly (by ∼25%) with 10-fold higher ONOOH/ONOO^−^ levels [68].

Exposure of human recombinant versican isoform V3 (rhV3) to ONOOH/ONOO^−^ resulted in a dose-dependent increase in non-reducible protein aggregates due, at least in part to intermolecular di-Tyr formation [89]. Nitration and hydroxylation of Tyr and Trp residues, and oxidation of Met residues, has been observed at 26 sites, with the most heavily nitrated site being Tyr161. This residue is located within the G1 domain, and the binding site for HA, with this resulting in reduced binding of HA to modified rhV3. Colocalization of versican and 3-nitroTyr has been detected in human atherosclerotic plaques [89].

The related PG aggrecan colocalizes with HOCl-generated epitopes in human atherosclerotic plaques [90]. Exposure of the G1-IGD-GD2 domains of aggrecan to HOCl (and reactive chloramines formed on amino acids, peptides, proteins and ECM) has been shown to result in site-specific fragmentation and aggregation of aggrecan. The precise sites of fragmentation have not been determined, but appear to lie within the inter-globular domain, and to be different from those generated by the protease ADAMTS1 [90].

### Isolated GAGs

Studies on the reactions of isolated GAGs with HO^•^, CO_3_^•−^, NO_2_^•^ and ONOOH/ONOO^−^ (both bolus, and formed time-dependently using the thermolabile compounds SIN-1) have provided evidence for polysaccharide backbone fragmentation, with this being dependent on the presence of O_2_ [91–94]. Gel electrophoresis experiments have provided evidence for the formation of ‘ladders’ with fragmentation at disaccharide intervals. Mechanisms to account for this effect have been proposed [91,92].

HOCl also induces depolymerization of GAGs but these reactions are mechanistically distinct from those described above, as the initial step involves formation of chloramines/chloramides at the amine/amide groups [95–97]. Subsequent reactions, and in some case dichloramine formation [97], result in site-specific fragmentation, with this involving radicals formed from the chloramines/chloramides in reactions involving O_2_^•−^ and O_2_ [95–100].

### Vascular cell-derived ECM

Various components within the ECM produced by HCAECs, HCASMCs and other cells, can be modified by HOCl, MPO-derived HOCl, and ONOOH/ONOO^−^. ECM species are over-represented among the modified proteins isolated from HCASMCs exposed to ONOOH/ONOO^−^ [69]. The effects of the modified matrices, generated by HOCl or MPO-derived HOCl, has been examined using naïve (non-oxidant exposed) HCASMCs, with this resulting in decreased adherence and a rounded cell morphology. The HCASMCs that adhered to the HOCl-modified ECM proliferated more than HCASMCs adherent to native ECM, and exhibited increased expression of mitosis-related genes. Other genes were also affected, with this including up-regulation of inflammatory genes (IL-6, IL-1β, and COX2), ECM genes (FN1 and LAMA1), and MMPs (MMP1, MMP11, and MMP13) [65].

ONOOH/ONOO^−^ -exposed HCAEC-ECM similarly results in decreased adhesion and spreading of naïve HCAECs. In these cells, gene expression was also altered, including changes to integrins and MMPs [67]. Exposure of naive HCASMC to ONOOH/ONOO^−^ modified ECM showed a biphasic cell adhesion response, with 1 µM ONOOH/ONOO^−^ resulting in enhanced cell adhesion, whereas higher levels of modifying agent gave decreased adhesion. This is possibly due to modification revealing additional binding sites at low oxidant concentrations, while cell-matrix interactions are lost upon exposure to higher oxidant doses. However, under the latter conditions increased proliferation and metabolic activity were detected, together with increased expression of genes involved in mitosis, ECM synthesis and inflammation (e.g. IL-1β, IL-6 and VCAM-1) [68]. These alterations have been interpreted in terms of a phenotypic switch of the HCASMC from a quiescent and contractile form to a proliferative and pro-inflammatory phenotype in response to the modified ECM, and an attempted remodeling of the modified ECM by the cells. Modulation of cell phenotype by modified ECM is not exclusive to vascular cells, with human lung fibroblasts also showing decreased cell adhesion and proliferation, and increased cytokine expression when cultured on HOCl and HOBr-modified ECM [101].

## Potential synergistic effects of protease and oxidative modifications

**Figure 4.**
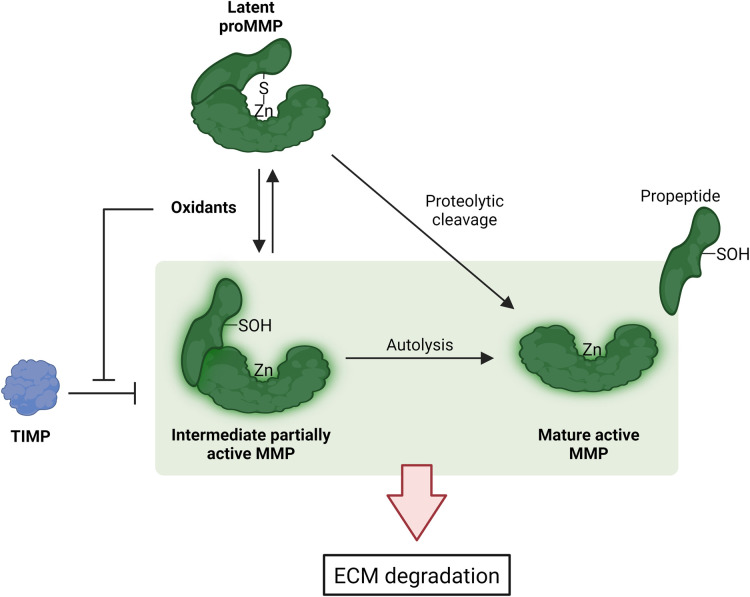
Mechanisms of MMP activation. Latent proMMP are maintained by co-ordination of the –SH of a conserved cysteine with the His-ligated zinc atom in the catalytic domain. The Zn-thiol bond can be disrupted by oxidants resulting in a partially active MMP (intermediate form) and subsequently becomes mature active MMP with the removal of the propeptide via autolysis. Alternatively the conversion of latent proMMP can be enzymatically activated to mature active MMP. MMP activity is inhibited by TIMP, which in turn is inactivated by oxidants. Created by the authors using Biorender.com.

MMPs, and related proteases have established roles in ECM degradation. MMPs are secreted as pro-forms that require activation. Studies have shown that oxidation can accomplish this, via oxidation of Cys73 of the pro-peptide, which normally binds to the active site zinc atom and thereby blocks the active site. Modification of this residue results in a loss of ligation, and generates of an active form of the enzyme (the ‘cysteine switch’ mechanism [102]). This can then undergo further processing to give the mature active enzyme, with both active forms of the enzyme capable of inducing further enzyme activation, and a ‘snowball’ effect of increasing activation (Figure 4). Such activation has been demonstrated for HOCl and MMP7 [103] and MMP9 [104], and also with other oxidants for MMP2 and MMP9 [105–107]. Furthermore, oxidants can inactivate the endogenous tissue inhibitors of these enzymes (TIMPs) [108,109], thereby amplifying the effects of enzyme activation and activity, and increasing ECM degradation. Similar processes may occur with other proteases.

## Consequences of ECM modification in atherosclerosis

A consequence of the above hypothesis that proteases and oxidation can act in concert and synergistically, is that there should be associations (and possible causal relationships) between the extents of oxidation, protease levels (and particularly *activity*) in human atherosclerotic plaques. There is abundant evidence for the presence of oxidized and other modified proteins and GAGs in human plaques, and also abundant evidence for elevated levels of many proteases, but direct evidence for links between these events, and causality is lacking. However, the *in vitro* studies reviewed above indicate that oxidants can both induce protease expression and activate these species. A recent study indicates a causal relationship between high neutrophil numbers and cardiovascular disease risk [110], with this likely to be due to enhanced intra-plaque inflammation, and increased MPO and protease activity. Furthermore, recent studies have provided robust proteomic data for different protein profiles (including MPO levels) between rupture-prone and stable human plaques [111], and for large numbers of fragmented proteins within rupture-prone human plaques (Lorentzen, submitted). These data highlight the important role of the ECM, and its integrity in plaque stability. Marked differential abundance of ECM- and associated proteins has been reported in plaques, with structural ECM proteins being more abundant in stable plaques, while proteins involved in ECM remodeling, protein degradation, and inflammation are enriched in rupture-prone plaques [111].

**Figure 5.**
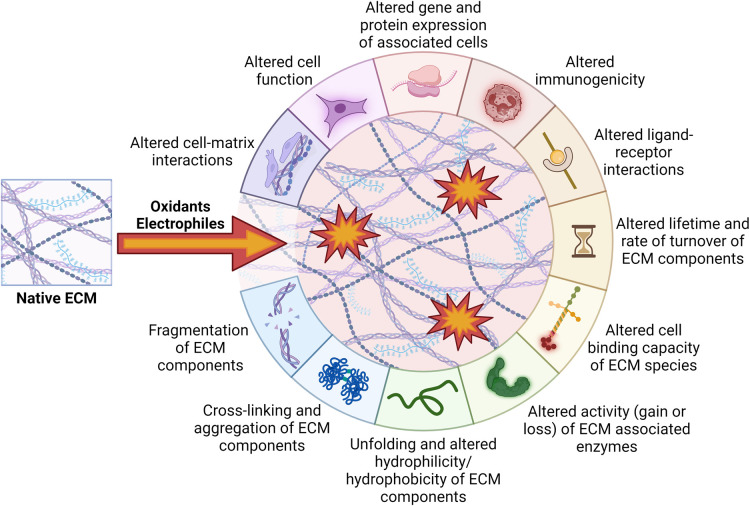
Functional consequences of modification of extracellular matrix species by oxidants and electrophiles. Exposure of native ECM to oxidants and electrophiles results in modification of the ECM. This can in turn affect cell-matrix interactions and cellular function. Created by the authors using Biorender.com.

GAGs in the subendothelial ECM can bind to Arg and Lys residues of apolipoprotein B-100 of LDL, thereby contributing to LDL accumulation. Modification of ECM components may result in changes in charge, hydrophilicity/hydrophobicity, reveal cryptic binding sites or induce cross-linking between ECM and LDL, thereby enhancing LDL retention. The protective fibrous cap of ECM components synthesized by VSMCs during the progression of atherosclerosis is important in plaque stability and the prevention of rupture. As highlighted above, modification by both electrophiles and oxidants can result in altered ECM structure, with this affecting binding and organization of ECM components as well as cell-matrix interactions, in turn altering cellular behavior (Figure 5). This may exacerbate EC dysfunction, induce pro-atherogenic phenotypic switching of VSMC, increase immune cell infiltration into the growing plaque (and thereby enhance oxidant- and electrophile levels), and also result (directly or through protease activation) in weakening of the ECM of the fibrous cap ECM, making it more rupture prone. Similar events may occur in other tissues, and further research is clearly needed to expand our understanding of the mechanisms at play.

## Conclusions

Current data indicate that ECM components, and particularly proteins, are major targets of PTMs, including oxidation, nitration and halogenation. Some of these appear to be physiological events, and of major functional importance. In contrast, others appear to be ‘bystander’ damage, and of potential pathological significance. The increasing sensitivity and sophistication of analytical methods is allowing increasing numbers and types of modifications to be detected including in ‘healthy’ samples, making the unraveling of positive (physiological) versus negative (pathological) events complex. In some cases this may be determined by their concentration rather absence/presence. Further work is clearly needed to unravel the role and significance of these events.

## Perspectives

- The ECM is a key regulator of tissue structure, function and integrity, with alterations to this being responsible for changes to cellular phenotype, function and activity.
- Multiple enzymatic and chemical reactions including those generated by oxidants and electrophiles can alter ECM components, with this resulting in changes to associated cell and tissue properties.
- Further research is needed to detect, quantify and characterize ECM changes during aging and disease. Understanding which modifications are intentional and beneficial, and which are damaging and deleterious, is critical to the development of targeted prevention and treatment strategies.

## Abbreviations

BM: basement membrane
CS: chondroitin sulfate
EC: endothelial cell
ECM: extracellular matrix
FGF2: fibroblast growth factor 2
FN: fibronectin
HA: hyaluronan
HCAEC: human coronary artery EC
HCASMC: human coronary artery smooth muscle cell
HOBr: hypobromous acid
HOCl: hypochlorous acid
HS: heparan sulfate
HUVEC: human umbilical vein endothelial cell
LDL: low-density lipoprotein
LN: laminin
LOX: lysyl oxidase
MDA: malondialdehyde
MMP: matrix metalloproteinase
MPO: myeloperoxidase
NC: non-collagenous
PDGF: platelet-derived growth factor
pFN: plasma fibronectin
PG: proteoglycan
PTM: post-translational modification
SMC: smooth muscle cell
TNF: tumor necrosis factor
VCAM-1: vascular cell adhesion molecule-1
VSMC: vascular smooth muscle cell
